# En Bloc Partial Laminectomy and Posterior Lumbar Interbody Fusion in Foraminal Spinal Stenosis

**DOI:** 10.4184/asj.2009.3.2.66

**Published:** 2009-12-31

**Authors:** Eung-Ha Kim, Hyung-Tae Kim

**Affiliations:** Department of Orthopedic Surgery, Soonchunhyang University College of Medicine, Bucheon, Korea.

**Keywords:** Lumbar vertebra, Spinal stenosis, Laminectomy, Posterior lumbar interbody fusion

## Abstract

**Study Design:**

A retrospective study.

**Purpose:**

An en bloc partial laminectomy and posterior lumbar interbody fusion (PLIF) in spinal stenosis patients with severe foraminal narrowing has a shorter operation time, less neural manipulation and allows indirect decompression by restoring the interforaminal height compared to other procedures. This study investigated the efficacy of the procedure.

**Overview of Literature:**

PLIF is one of the most popular surgery for degenerative spine such as foraminal spinal stenosis, instability spondylolisthesis and discogenic pain. Various techniques for PLIF have their own advantages and disadvantages. But in some severe cases, we need an efficient method of PLIF for decompression and fusion.

**Methods:**

This study examined 61 patients, who had 85 levels treated with PLIF using an en bloc partial laminectomy and facetectomy, and could be followed up for more than 2 years. The mean age of the patients and mean follow up period was 66 years and 39 months, respectively. The clinical results were evaluated using the MacNab's criteria, Visual Analogue Scale (VAS) score, and Korea Version Oswestry Disability Index (KODI). The union of the intervertebral space was evaluated using Lenke's criteria. The intervertebral angle and height of the posterior intervertebral disc were also measured.

**Results:**

Excellent and good results were obtained in 54 cases (89%) according to MacNab's criteria. The VAS and KODI scores were 8.1 and 34.6, preoperatively, and 3.4, and 14.1, postoperatively. Bone union was A and B grades according to Lenke's criteria in 57 cases. The mean segmental angle and mean height of the posterior disc were respectively, 7.4° and 6.5 mm preoperatively, 9.1° and 10.6 mm postoperatively, and 8.0° and 9.7 mm in the last follow-up. There were 5 cases of postoperative infection, 4 cases of junctional problems and 1 case of screw malposition.

**Conclusions:**

En bloc partial laminectomy and PLIF is an effective method for treating severe spinal stenosis with foraminal narrowing.

## Introduction

Decompression is a standard treatment regimen for the surgical treatment of lumbar spinal stenosis, and fusion is required in many cases after extensive decompression^1^,^2^. In particular, an extensive facetectomy is needed for decompression of the foraminal stenosis in many cases. In foraminal decompression, there is a possibility of nerve root injury due to the retractor or a narrow surgical field. In cases with a remaining facet, a cage should be inserted with the nerve root retracted, which also suggests the possibility of nerve root injury due to excessive traction. In cases of lumbar spinal stenosis with concomitant foraminal stenosis, an en bloc partial laminectomy can shorten the surgical time, minimize the manipulation of the nerve root and minimize the bone loss for a local bone graft. In cases of spinal stenosis requiring fusion, the posterior lumbar interbody fusion theoretically resolves the instability after decompression and also widens the foraminal space. This makes it possible to indirectly decompress the foraminal stenosis, correct the deformity and relieve the discogenic back pain^3^,^4^. For the surgical treatment of spinal stenosis that requires decompression and stabilization, posterior lumbar interbody fusion using an en bloc partial laminectomy allows complete decompression compared to the conventional type of laminectomy using a punch. A secure and wide surgical vision can minimize the need for neural manipulation. The grafted local bone obtained during decompression can also reduce the bone loss. This study evaluated the efficacy of posterior lumbar interbody fusion using an en bloc partial laminectomy.

## Materials and Methods

### 1. Subjects

Sixty one cases (87 segments), who underwent posterior lumbar interbody fusion using en bloc partial laminectomy between May of 2004 and February of 2007, and could be followed up for more than 2 years, were enrolled in this study. There were 24 men and 37 women. Patients with lumbar spinal stenosis or pseudospondylolithesis, who concurrently had a foraminal stenosis were indicated. There were 35 cases of 1 segment and 26 cases of 2 segments. Of these, 36 segments also had pseudospondylolithesis. Surgery was performed by the author in a single-institution setting. The mean age of the patients was 66 years (range, 45 to 82 years) and the mean follow-up period was 39 months (range, 24 to 57 months). In this series, the surgical indications included the following:

1) Extensive decompression was required due to the presence of severe foraminal stenosis.
2) There was instability or deformity due to foraminal stenosis and a chief complaint of discogenic back pain.
3) Degenerative spondylolisthesis with a current foraminal stenosis.

### 2. Surgical procedure

**Fig. 1:**
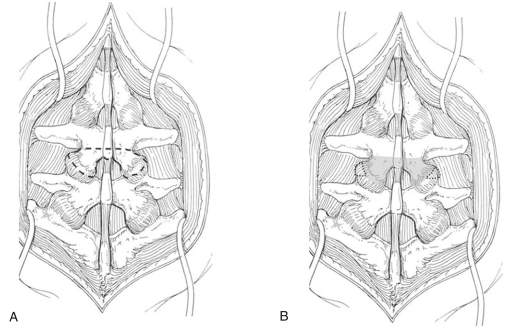
(**A**) En bloc laminectomy with inferior articular processes. The area designated by the dashed line was excised. (**B**) Osteotomy of the superior halves of the superior articular processes. The area designated by the dotted line was excised.

The conventional paraspinal posterior approach was used. But we did not need to exposure whole transverse process. In regard to an en bloc partial laminectomy, the inferior 1/2 of lamina and inferior 1/2 of the spinous process were resected using a power saw. 1/4 inch osteotome was then used to complete the osteotomy by tap and twisting motion. Ligamentum flavum was detached from the lamina using a curette. The posterior facet capsule was removed. The resected lamina containing the inferior articular process was removed en bloc from the underlying dura (Fig. 1A). The superior articular process of the inferior segment was resected using a power saw and an osteotome up to the superior level of the pedicle. The resected facet fragments were elevated and removed using a small-sized curette and rongeur. Foraminal decompression was performed and the disc was exposed to the lateral side (Fig. 1B). Bleeding control could be facilitated under wide surgical vision. For posterior lumbar interbody fusion, the annulus fibrosis was resected laterally from the just lateral side of dura in a rectangular shape. The intervertebral space was sequentially distracted using an intervertebral body distractor. At this time, the nerve root was easily protected without medial retraction of the dura. The superior and inferior endplate of the adjacent vertebral body were prepared by ring curette. The cage (manufacturer U & I, Neo-IC cage, in most cases, length 24 mm, height 10 mm, slope angle 4°) was filled with autogenous local bone that had been obtained from the resected lamina. The cage was inserted into the intervertebral space and rotated 90°. Therefore, the intervertebral height and lordotic angle were restored. Foraminal decompression was sufficient without an additional foraminotomy, which was confirmed by probing. The fusion was stabilized using a pedicle screw. All patients wore a thoracolumbosacral orthosis for six weeks. Ambulation was allowed on postoperative 1 day after removing the closed suction drain and standing exercise.

### 3. Evaluation

For a clinical evaluation, the clinical records, operation record and outpatient clinic record at the final follow-up were examined, and a telephone interview was performed. The Macnab classification system (Table 1), Visual Analogue Scale (VAS) score of the preoperative and postoperative back pain, lower leg radiating pain, and Korea Version Oswestry Disability Index (KODI) were used. The diagnosis was made based on the physical findings, radiological evaluation and MRI. Fusion was evaluated by plain radiographs according to Lenke's criteria^5^. The changes in the segmental angle and the posterior disc height were also measured.

Statistical analysis was performed using SPSS ver. 12.5 (SPSS Inc., Chicago, IL, USA). The preoperative and postoperative findings were compared based on the clinical and radiological outcomes. A paired t-test was used to compare the VAS, KODI, segmental angle and posterior disc height between the two groups. A p-value<0.05 was considered significant.

## Results

### 1. Clinical outcomes

Fifty four out of the 61 cases (89%) achieved more than good according to the Macnab criteria. Four (6%) and 3 (5%) cases had fair and poor clinical outcomes, respectively (Table 2). The postoperative back pain and lower leg radiating pain decreased from 8.1 points (range, 6 to 10 points) to 3.4 points (range, 0 to 10 points) (p=0.034). The KODI decreased from 34.6 points (range, 29 to 40 points) to 14.1 points (range, 0 to 35 points; p=0.028) (Table 3). Of the seven cases with fair or poor results, there were 2 infections, 1 non-infective pseudoarthrosis, 1 adjacent segment problem, 1 deep vein thrombosis, 1 cauda equina syndrome, and 1 compensatory action associated with the litigation problem. Two infections were treated surgically for postoperative infectious non-union. Bony union was achieved with an autologous bone graft in 1 case after removing the screw and cage. Regarding the adjacent problem, the lower leg radiating pain was resolved gradually with conservative treatment.

### 2. Radiological outcomes

According to Lenke's criteria, 31, 26, and 4 cases were graded as Grade A, B, and other Grades, respectively. There were 2 cases of non-union due to postoperative infections. One non-union was to the result of a postoperative infection and was treated by removing the pedicle screw and the cage, followed by an autogenous bone graft. The other nonunion was managed by removing the cage and autologous bone graft. There was 1 case of non-infectious non-union, in whom the symptoms were improved by posterolateral fusion. The symptoms in the remaining case of adjacent problems were improved by conservative treatment. The mean segmental angle was increased from 7.4° (range, 3.2° to 12°) preoperatively to 9.1° (range, 7.2° to 14.1°) postoperatively (p=0.024). At a final follow-up, the mean segmental angle was 8.0° (range, 6.3° to 12.8°). The mean posterior disc height increased from 6.5 mm (range, 1.5 to 13.4 mm) preoperatively to 10.6 mm (range, 8.7 to 13.5 mm) postoperatively (p=0.023). The mean posterior height at the final follow-up was 9.7 mm (range, 5 to 12.7 mm) (Table 3).

### 3. Complications

There were 5 cases of postoperative deep infection, 1 noninfective pseudoarthrosis, 4 adjacent problems, 1 screw malposition, 1 deep vein thrombosis, 1 cauda equine syndrome, and 4 dura tears.

## Discussion

An en bloc partial laminectomy and posterior lumbar interbody fusion yielded satisfactory outcomes in 54 of the 61 cases. Of the seven cases with fair or poor treatment outcomes, two cases had a non-spinal causative factor. This procedure could be extended multilevels, but our cases here were operated at 1 or 2 segments.

A different surgical approach is needed for the surgical treatment of spinal stenosis depending on the severity of the stenosis and the presence of instability. A simple partial laminectomy or partial facetectomy can improve the symptoms. However, in many cases in whom the stenosis was extensive or extended to the intervertebral foramen or its lateral side, a complete decompression cannot be obtained without sacrificing of the facet. Furthermore, bony fusion would yield a better treatment outcome in cases with concurrent instability, or potential discogenic back pain^4^,^6^-^8^. In the current procedure, an extensive laminectomy provided the surgical ease for posterior lumbar interbody fusion, and the wider surgical vision allowed easy bleeding control. In cases of severe spinal stenosis involving the intervertebral foramen, minimally invasive spinal surgery cannot achieve complete decompression due to the restricted extent of the surgical vision. Within the narrow space, severe nerve injury might occur during posterior lumbar interbody fusion. In addition to the technical difficulty and prolonged surgical time, unlike the traditional treatment regimens, it is not easy to identify the problems and to resolve them immediately, even though some problems might occur during surgery. Foley et al.^9^ reported that a mean surgical time of 290 minutes was required for minimal invasive posterior lumbar interbody fusion and percutaneous pedicle screw fixation in 15 patients. According to Park et al.^10^, through a comparison of degenerative lumbar disease between minimal invasive spinal surgery and the traditional approach, after minimal invasive spinal surgery, the mean surgical time was 216.5 minutes in ten early-stage cases and 181.5 minutes in ten late-stage cases. They reported that the minimally-invasive surgical procedure was difficult and it took longer to become acquainted with the surgical technique compared to the traditional approach. In addition, Podichetty et al.^11^ performed minimal invasive spinal decompression in 220 patients with spinal stenosis. According to these authors, there were complications, such as postoperative infections and facet fractures, in 5.9% of patients. According to Khoo and Fessler^12^, back pain was completely resolved in 16% of patients and the symptoms were improved in 68% of patients. However, they noted that there were no changes in the symptoms in 16% of cases. In patients requiring extensive decompression due to the presence of severe foraminal stenosis, effective decompression was time consuming and narrow vision using the conventional laminectomy. In cases of spinal stenosis, the current procedure removed the lamina promptly using an en bloc procedure, which is in contrast to that using other palliative laminectomy or minimal invasive spinal decompression. Therefore, complete decompression was possible within the shortest time possible. The area where the nerve was compressed was easily confirmed. Therefore, it was assumed that it reduces the unnecessary neural manipulation and can also perform the decompression. In addition, the degree of neural manipulation was relatively lower and the intervertebral space was reconstructed because a cage was inserted in the area where a facetectomy had been performed. The vertebral foramen was decompressed indirectly. Hence, a complete, convenient foraminal decompression might be possible.

There is some controversy regarding fusion after decompression. It was reported that bone fusion after a laminectomy produced good treatment outcomes^1^,^2^. In cases with concurrent instability or facet removal, pedicle screw stabilization has been established as a standard treatment regimen. However, there is considerable controversy as to whether it is a posterolateral fusion or a posterior lumbar interbody fusion. On a theoretical basis, posterior lumbar interbody fusion was close to the central axis of this loading. Therefore, it was an ideal area for bone fusion. By restoring the intervertebral disc height, there is an advantage that the deformity can be corrected and the intervertebral foraminal stenosis can be decompressed indirectly. However, there is a higher likelihood of neural injury or bleeding compared to the posterolateral fusion^8^. Our procedure was intended to minimize these disadvantages of posterior lumbar interbody fusion. It also assumed that there are additional advantages of a shorter surgical time and minimal graft bone loss.

In cases of degenerative spondylolisthesis, only a laminectomy has been performed. Since the 1980s, fusion has been reported to gradually produce good treatment outcomes. After a simple decompression, bone ingrowth occurs in the resected lamina. Of these, many parts have been reported to show a recurrence of symptoms^3^,^13^,^14^. According to Katz et al.^15^, revision surgery was performed in 17% of patients after the laminectomy due to the occurrence of instability and restenosis. In 30% of patients, severe back pain was reported to remain. There are several reports of the treatment outcomes of posterior lumbar interbody fusion performed in cases of degenerative spondylolisthesis. Bridewell et al.^16^ reported that the fusion rate was improved using a pedicle screw, and better clinical outcomes were obtained after a more than two years follow-up of 44 patients with degenerative spondylolisthesis. Nah et al.^8^ examined 40 cases in whom a more than two years follow-up study was possible for the management of 4-5th lumbar posterior lumbar interbody fusion. Twenty one cases had only posterolateral fusion performed and 19 cases underwent additional posterior lumbar interbody fusion. They showed that the posterior lumbar interbody fusion produces excellent clinical outcomes if the preoperative instability is of a greater degree. Shin et al.^17^ performed a follow-up over a 10-year period of 44 cases in whom lumbar interbody fusion was performed for the treatment of degenerative lumbar disease. According to these authors, pedicle screw loosening and breakage occurred in three cases. In seven cases (12%), revision surgery was performed due to the adjacent segmental problems. It was assumed that the current procedure would be effective considering the clinical value of posterior lumbar interbody fusion in cases of degenerative spondylolisthesis, in which there is the concomitant presence of foraminal stenosis. In most cases of degenerative spondylolisthesis, there is the concurrent presence of severe stenosis and severe facet hypertrophy. Using this method, we were successful in shortening the time for decompressing this area. In the current cases, there was the concomitant presence of degenerative spondylolisthesis in 36 segments. In cases of foraminal stenosis and instability, neural manipulation could be minimized and both complete decompression and posterior lumbar interbody fusion could also be performed using the current procedure.

An en bloc partial laminectomy and posterior lumbar interbody fusion have the following advantages:

1) The decompression time can be shortened.
2) During the decompression, neural manipulation can be reduced.
3) A posterior lumbar interbody fusion can be performed under good surgical vision with less neural manipulation.
4) A graft bone loss can be minimized.

At the preoperative planning stage, we could expect the parts of the lamina and posterior facet that should be resected if a wide decompression was necessary. Therefore, the surgical time can be reduced by en bloc partial laminectomy of this portion. During the decompression procedure, the unnecessary manipulation of the neural tissue was reduced. After inserting a cage in the intervertebral body, attempts were made to indirectly decompress the intervertebral foramen. There might be an advantage of posterior lumbar interbody fusion where the deformity can also be corrected. It can be confirmed whether the nerve root can be decompressed if the disc can be exposed sufficiently to the extralateral side. Hemostasis was achieved after a wider surgical vision. Without an excessive traction of the dura, a cage can be inserted for posterior lumbar interbody fusion. In determining the scope of decompression, some areas for which en bloc laminectomy is considered to be excessive can be included. However, in the current cases, partial conservation was not considered to be helpful for preventing the instability. There were advantages due to the acquisition of a wider surgical vision, such as the complete achievement of decompression, accurate bleeding control, less neural manipulation for the insertion of a cage and a prompt surgical procedure. The lamina that was removed en bloc was used as graft bone without bone loss in the cage. The allogeneic bone graft was performed using an additional intervertebral graft bone. A cage was lifted up in a 90° rotation and had a lordotic angle of 4°. There are some cases in whom it was effective for partially restoring lordosis. In some cases, restoration of lordosis was not effective due to some factors such as location of the cage, shape of endplate and osteoporosis. The mean level of recovery of the posterior disc height, which is considered to be one of the indicators for the decompression of intervertebral foramen, was approximately 4 mm. The level of recovery was well maintained at the follow-up study. Both clinically and radiologically, satisfactory outcomes were obtained using an en bloc partial laminectomy and posterior lumbar interbody fusion. It is believed that the current procedure will be very useful for suitable patients.

With regard to complications, there were five cases of infection, which is a relatively high incidence. However, the occurrence of infection was crowded at a certain period. In three cases, the symptoms were improved using IV antibiotics therapy. In two cases, additional surgery was required, which included the removal of the internal device or curettage. An adjacent problem (the degenerative changes of adjacent segments) was encountered in four cases. However, the corresponding cases were followed up using the conservative treatment due to the unclear presence of back or lower leg radiating pain with symptomatic improvements in 3 cases. One case of cauda equine syndrome occurred due to compression arising from a postoperative extradural hematoma. The hematoma was removed the next day after surgery. Two years after surgery, there were no problems with ambulation but the patient presented with bladder dysfunction. There were four cases of mild dura tear all of them were identified at operative field and managed with watertight sutures and fibrin glue without any continual CSF leakage.

This study had some limitations. No groups could be compared due to the lack of a control group, and the follow-up period was relatively short. According to Postacchini et al.^18^,^19^, at an 8.2-year follow-up carried out on 64 patients who were treated surgically for spinal stenosis, satisfactory treatment outcomes were obtained in 67%. They also noted that satisfactory treatment outcomes were obtained in 79% of 54 patients who were satisfied with the treatment outcomes on the first postoperative year. According to Katz et al.^20^, at a 7- to 10-year follow-up, revision surgery was required in 23% of their 88 patients with spinal stenosis who underwent fusion or not after decompression. They noted that satisfactory treatment outcomes were obtained in 75% of subjects. It has been acknowledged that the satisfaction of patients deteriorates gradually with time. Therefore, a long-term follow-up will be needed for patients in whom the current procedure had been performed.

## Conclusions

Posterior lumbar interbody fusion using an en bloc partial laminectomy is an effective surgery for patients with concomitant degenerative spinal stenosis and foraminal spinal stenosis where wide decompression and fusion is needed. However, long-term follow-up controlled trials on a larger scale using conventional methods and minimal invasive spinal surgery will be needed to confirm the efficacy of posterior lumbar interbody fusion using an en bloc partial laminectomy.

## Figures and Tables

**Table 1:**
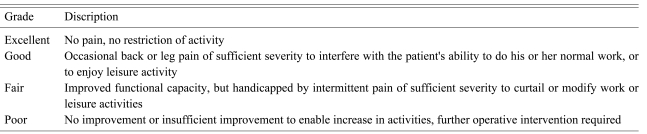
Macnab classification

**Table 2:**
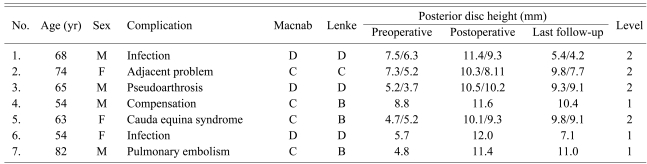
Fair & Poor result cases after en bloc laminectomy & posterior lumbar interbody fusion (PLIF)

Posterior disc height was designated by one number for 1 level case, and 2 numbers for 2 level cases.

**Table 3:**
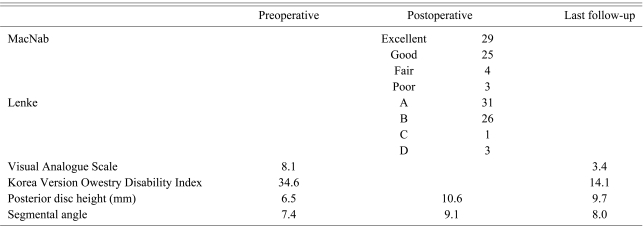
Clinical and radiological results
